# Editorial: Sustainable Catalytic Production of Bio-Based Heteroatom-Containing Compounds

**DOI:** 10.3389/fchem.2020.628859

**Published:** 2020-12-11

**Authors:** Hu Li, Song Yang, Yaqiong Su

**Affiliations:** ^1^State Key Laboratory Breeding Base of Green Pesticide and Agricultural Bioengineering, Key Laboratory of Green Pesticide and Agricultural Bioengineering, Ministry of Education, State-Local Joint Laboratory for Comprehensive Utilization of Biomass, Center for R and D of Fine Chemicals, Guizhou University, Guiyang, China; ^2^Laboratory of Inorganic Materials and Catalysis, Schuit Institute of Catalysis, Eindhoven University of Technology, Eindhoven, Netherlands

**Keywords:** sustainable chemistry, biomass conversion, catalytic mechanism, biorefinery, biofuels

Organic synthesis is a versatile tool in the design and creation of desirable scaffolding molecules. These processes typically involve toxic and non-renewable starting materials as well as the formation of waste or byproducts. Biomass, on the other hand, is deemed as the most abundant organic carbon resource, and the development of sustainable approaches to producing valuable organic molecules from waste and biomass feedstocks is of great significance for alleviating environmental pollution, deterioration, and the greenhouse effect.

One of the prominent features of biomass is that it is rich in oxygen, the compounds it yields are typically functionalized with oxygen-containing species such as hydroxy, ether, carbonyl, carboxyl, and ester groups, which significantly enrich the product variety. Derived from these oxygen-rich bio-products, the catalytic functionalization of biomass derivatives with heteroatoms such as nitrogen, sulfur, phosphorus, and silicon can also be realized via specific reaction routes or pathways. These functionalized compounds are crucial core scaffolds or key intermediates in a wide range of pharmaceutical molecules, fiber dyes, and printing ink, which can also be directly used as solvents, surfactants, and so on.

This Research Topic presents a collection of original research and review articles on bio-based heteroatom-containing compounds, including green and facile production of biodiesel (Zhang et al.; Zhu et al.), chemical synthesis, structure, and the evaluation of the bioactivities of naturally occurring haedoxan-like molecules (Chen et al.), pyridylpyrazolamide derivatives containing pyrimidine motifs (Wu et al.), and bioflavonoid-metal complexes (Yao et al.). The Research Topic provides insights into the effect of Ni-ZSM-5 catalysts on biomass pyrolysis (Ding et al.), and into the catalytic strategies developed for depolymerization of cellulosic biomass to value-added products (Luo et al.; Liu et al.). The Research Topic also depicts the process of doping heteroatom in phosphor to improve the resulting luminescent properties of the material (Deng et al.).

An original research paper by Zhang et al. reports a simple and low-cost impregnation method for the facile preparation of metal-organic framework Cu-BTC-supported Sn (II)-substituted Keggin heteropoly nanocomposites (Sn1.5PW/Cu-BTC). The obtained Sn1.5PW/Cu-BTC nanocatalyst show high activity in the production of biodiesel from oleic acid through esterification, with relatively low apparent activation energy (*Ea*) of 38.3 kJ mol^−1^, and can be reused seven times with no significant loss of activity. Zhu et al. disclose that Donor-Acceptor (D-A) cyclopropanes can react with α,β-unsaturated enamide substrates via chemo- and diastereoselective (3 + 2) cycloaddition reaction under basic conditions for efficient access to spiro (cyclopentane-1,3'-indoline) derivatives, in which green, inexpensive, and readily available NaOH were used as the sole catalyst to promote this transformation. The authors expand a broad range of D-A cyclopropanes as the C-3 synthons to react with oxindole-derived α,β-unsaturated enamides, furnishing structurally sophisticated spiro(cyclopentane-1,3'-indoline) derivatives with up to 3 adjacent chiral centers in excellent yields as single diastereomers.

The work of Wu et al. reveals the antifungal and insecticidal activities of pyridylpyrazol amide derivatives containing a pyrimidine moiety, synthesized via six-step reactions, including hydrazidation, cyclization, bromination or chlorination, oxidation, hydrolyzation, and condensation. The antifungal properties of some title compounds against *Sclerotinia sclerotiorum, Phytophthora infestans, Thanatephorus cucumeris, Gibberella zeae, Fusarium oxysporum, Cytospora mandshurica, Botryosphaeria dothidea*, and *Phompsis* sp. are similar to those of Kresoxim-methyl or Pyrimethanil at 50 μg/mL. These synthesized compounds show a certain insecticidal activity against *Spodoptera litura, Mythimna separata, Pyrausta nubilalis, Tetranychus urticae, Rhopalosiphum maidis*, and *Nilaparvata lugens* at 200 μg/mL. Yao et al. evaluate the general synthetic procedures for bioflavonoid–metal complexes that are unstable in air-saturated alkaline solutions. All examined bioflavonoid–metal complex ligands (e.g., dihydromyricetin, myricetin, quercetin, daidzein, genistein, chrysin, baicalein, rutin hydrate, and kaempferol) dissolved in air-saturated alkaline solutions can generate ${\text{O}}_{2}^{-}$ at different capacities, as demonstrated by electron paramagnetic resonance (EPR) analysis, indicating that the general procedures for the synthesis of bioflavonoid–metal complexes using a transition metal ion and an air-saturated alkaline solution may require improvement. Deng et al. uncover that doping GdAlO_3_ phosphors with Er^3+^/Yb^3+^/Tm^3+^, which were prepared by the co-precipitation method, can effectively adjust the up-conversion light performance, especially the white-emitting luminescence properties.

This Research Topic features several review articles with distinct scopes (Ding et al.; Luo et al.; Liu et al.; Chen et al.). Ding et al. mainly review the research progress of ZSM-5 zeolite supported Ni (Ni-ZSM-5) catalysts in pyrolysis and hydro-pyrolysis of biomass, including (i) the single metal Ni-ZSM-5-enabled catalytic conversion of biomass in the absence of hydrogen, (ii) Ni-ZSM-5-promoted biomass catalytic conversion in a hydrogen atmosphere, and (iii) biomass valorization using ZSM-5 supported bimetal catalysts composed of Ni and other metals. The authors focus on the recent investigation of Ni-modified microporous ZSM-5 materials used in catalytic pyrolysis of lignin and cellulose, covering applications of metal-modified hierarchical ZSM-5. Luo et al. provide a comprehensive view on state-of-the-art heteropoly acid (HPA)-based catalysts for the hydrolytic depolymerization of cellulosic biomass. With unique properties such as good solubility, high thermal stability, and strong acidity, HPA-based catalysts are revealed to be efficient for depolymerization and conversion of cellulose into valuable chemicals and biofuels, which is one of the most remarkable processes in chemistry for sustainability. The authors summarize the characteristics, advantages, and applications of HPAs in different categories for cellulose degradation and discuss the mechanisms of HPAs catalysts in the effective degradation of cellulosic biomass, which provides more avenues for the further development of renewed and robust HPAs utilized for cellulose degradation. Liu et al. illustrate the effects of biomass pretreatment method, catalyst texture/acidity, involved catalytic mechanisms, and different possible intermediates (e.g., diethyl ether, 4,5,5-triethoxypentan-2-one, ethoxymethylfuran, ethyl-D-fructofuranoside, and ethyl-D-glucopyranoside) on the catalytic transformation of biomass saccharides into alkyl levulinates (ALs), which have widespread applications like fuel additives, flavorings, plasticizing agents, and synthetic precursors to various building blocks. The authors disclose several typical conversion processes or routes for the synthesis of ALs from renewable resources, mainly including (i) direct esterification of levulinic acid with alkyl alcohols and (ii) alcoholysis of relevant biomass feedstocks, such as furfuryl alcohol, chloromethyl furfural, and saccharide. Chen et al. briefly introduce the chemical structures of naturally occurring haedoxan-like molecules that exhibit promising insecticidal, antifungal, antibacterial, and anticancer activities. The authors detail the synthetic efforts toward haedoxans and phrymarolins in the past three decades.

This Research Topic intends to enlighten researchers about more eco-friendly and sustainable synthetic procedures, shedding light on renewed catalytic strategies and routes developed for the production of bio-based heteroatom-containing compounds, indicating the enthusiasm and commitment of researchers working in this area.

## Author Contributions

All authors made a substantial, direct, intellectual contribution to the work, and approved it for publication.

## Conflict of Interest

The authors declare that the research was conducted in the absence of any commercial or financial relationships that could be construed as a potential conflict of interest.

